# National Hospital for the Paralysed and Epileptic

**Published:** 1907-03-16

**Authors:** 


### NATIONAL HOSPITAL FOR THE PARALYSED AND EPILEPTIC.

### SIR EDGAR SPEYER ON ELECTRICITY AND ITS EFFECTS ON MODERN LIFE.

Sir Edgar Speyer presided at the festival dinner of the National Hospital for the Paralysed and Epileptic, which was held at the Whitehall Rooms on Monday, March 11. Sir Edgar Speyer said that it had required all his courage and a very thick skin to act as chairman for that dinner, because he did not think it would have been possible to have chosen a more unfavourable year for a begging expedition through the City, which was his usual happy hunting ground, than the present one. To beg was not a pleasant calling, and he was not a good beggar. Hospitals like that for which he was appealing did not necessarily appeal to all. Those who had children naturally took an interest in children's charities. Those who had lost dear friends and relations from those scourges cancer and consumption felt called on to assist institutions which were engaged in fighting those diseases. The National Hospital, however, dealt with a class of disease which was, from every point of view, except, perhaps, the scientific, unpopular. It did not appeal to the gallery, if he might so express it. Diseases of the brain and the spinal cord were of all diseases most difficult to deal with, troublesome to relatives, and often not even interesting to an amateur nurse. (Laughter). Take the case of the epileptic. He is often, in the intervals between the alarming fits to which he was subject, apparently quite well, and his appetite was as good as his fellows—or better. Yet he could not go out to earn his living as others did, and his disease was frequently accompanied by characteristics which added to his unpopularity. He was often morose, untruthful, and unreliable, and he was far less likely to be tenderly treated. Then, too, a man might suddenly be struck down by paralysis and be at once deprived of the use of a limb for an indefinite period. When his hearers remembered what that meant in their own homes they would realise what it was in a family of poor people, whose home might not be too fit even for the proper accommodation of the healthy. To provide for these was only a part of the good work which was being done in the National Hospital; for, besides this, the hospital had as its aim the cure of nervous diseases. This age had been called the age of nerves; and there could be no doubt that the present-day life, with its keen competition, its hurry, and its bustle caused by the modern means of communication, meant great strain on the nervous system. The present-day conditions rendered proper change from work to recreation harder to achieve, and the consequence was an annual increase in sufferers from diseases of the nerves and brain. As one who had taken a fair share in the extension of modern methods of locomotion in London, the thought had often struck him that in providing for what he had considered an obvious necessity for London he was indirectly increasing that wear and tear of life which was the cause of nervous diseases. But at the same time it would indeed be a strange—nay, a providential—act of nature if electricity, which had given them electric traction, telegraphs, and nerve-racking telephones, should also prove, as he believed it was in a large measure proving to be, the cure of those diseases which were often the outcome of pressure and long-continued nervous irritation. (Hear, hear.) At any rate, great progress had been made, and chiefly through the work of the National Hospital, in their knowledge and cure of nervous diseases; and it was on behalf of both the investigation and the cure of these diseases that he appealed to his hearers, and through them to a wider public, that night. Cure and prevention went hand in hand in the National Hospital, its staff was renowned far beyond the boundaries of even Great Britain, and it contained names which were household words all over the world. After alluding to the great pressure on the hospital both as regards the wards and the out-patients' department, the Chairman said he was one of those who believed that to manage a charity required the same qualities, the same attention to detail, the same strict adherence to well-considered economy, as did any business, and he ventured to think that the cause of true charity was to a large extent frustrated if those principles were not applied. In the case of the National Hospital he was fully satisfied that under the able chairmanship of Mr. Macmillan sound business methods held the scales evenly between sympathy and common sense, and that the money was well and economically spent.

Other toasts followed, and the Secretary announced that the Festival Dinner had resulted in a collection of £2,481, of which £2,123 was given to the hospital and £358 to the Research Fund.

